# Supplementary material for: Discrepancies in Persistent Dry Eye Signs and Symptoms in Bilateral Pseudophakic Patients
Source: J Clin Med. 2019 Feb 7;8(2):211. doi: 10.3390/jcm8020211 (PMC6406599; doi:10.3390/jcm8020211)
Supplement: Supplementary file 1 [file jcm-08-00211-s001.pdf]

Table S1. Univariable and multivariable logistic regression analyses of dry eye subjective symptoms and objective signs in relation to the history of cataract surgeries in both sexes.

	Men				Women			
**Characteristics**	**Univariable OR (95% CI)**	***P* Value**	**Multivariable OR (95% CI)** **	***P* Value**	**Univariable OR (95% CI)**	***P* Value**	**Multivariable OR (95% CI)** **	***P* Value**
**Subjective Symptoms**								
Dry sensation	1.02 (0.44–2.38)	0.95	1.00 (0.40–2.55)	0.98	0.63 (0.40–0.99)	0.05	1.04 (0.63–1.72)	0.88
Foreign-body sensation	1.72 (0.76–3.87)	0.19	1.09 (0.44–2.71)	0.85	1.03 (0.65–1.61)	0.91	1.02 (0.63–1.66)	0.93
Ocular pain	0.94 (0.12–7.57)	0.95	1.79 (0.19–17.1)	0.61	0.77 (0.36–1.64)	0.50	0.85 (0.38–1.91)	0.70
Ocular fatigue	0.98 (0.51–1.89)	0.95	1.19 (0.58–2.44)	0.63	0.74 (0.49–1.12)	0.16	0.94 (0.60–1.47)	0.79
Sensitivity to bright light	0.92 (0.38–2.24)	0.85	0.89 (0.34–2.32)	0.80	0.54 (0.31–0.95)	0.03	0.49 (0.27–0.88)	0.02
Blurred vision	1.26 (0.63–2.55)	0.51	0.88 (0.41–1.89)	0.74	0.83 (0.50–1.39)	0.49	0.76 (0.44–1.31)	0.32
**Objective Signs** †								
Short tear break-up time	1.59 (0.91–2.78)	0.11	1.36 (0.74–2.50)	0.32	1.06 (0.71–1.58)	0.80	1.17 (0.76–1.79)	0.48
Corneal epitheliopathy	1.96 (0.98–3.93)	0.06	1.93 (0.88–4.23)	0.10	1.41 (0.94–2.12)	0.10	1.53 (0.98–2.39)	0.06
Short maximum blinking interval	1.50 (0.80–2.83)	0.21	0.90 (0.44–1.82)	0.77	1.28 (0.86–1.90)	0.22	1.00 (0.65–1.52)	0.98

† Abnormal ocular surface parameters were defined as follows: tear break-up time ≤5 s, the presence of corneal epitheliopathy (keratoconjunctival staining score ≥3 points according to the Japanese dry eye criteria), and maximum blinking interval <10 s. ** Adjusted for age (in 10-year age groups), sex (male vs. female), and the use of dry eye medications (yes vs. no). Abbreviations: CI: confidence interval; OR: odds ratio.

Table S2. Association between maximum blinking interval and other dry eye objective signs in the no subjective dry eye symptoms strata *

	Maximum Blinking Interval					
	**Dry Sensation (-)**	**Foreign-Body Sensation (–)**	**Ocular Pain (–)**	**Ocular Fatigue (–)**	**Sensitivity to Bright Light (–)**	**Blurred Vision (–)**
**Characteristics**	**Spearman Correlation Coefficient (*P* Value)**					
**Tear Break-Up Time**						
No surgery	0.28 (<0.001)	0.30 (<0.001)	0.27 (<0.001)	0.26 (<0.001)	0.28 (<0.001)	0.26 (<0.001)
Postcataract surgeries	0.24 (0.004)	0.30 (<0.001)	0.27 (<0.001)	0.27 (0.003)	0.26 (0.001)	0.23 (0.005)
**Keratoconjunctival Staining Score**						
No surgery	−0.15 (<0.001)	−0.18 (<0.001)	−0.15 (<0.001)	−0.16 (<0.001)	−0.15 (<0.001)	−0.16 (<0.001)
Postcataract surgeries	−0.14 (0.10)	−0.17 (0.05)	−0.18 (0.02)	−0.26 (0.004)	−0.22 (0.007)	−0.16 (0.05)

* Spearman correlation coefficients between maximum blinking interval and tear break-up time or keratoconjunctival staining score.
